# IV-YOLO: A Lightweight Dual-Branch Object Detection Network

**DOI:** 10.3390/s24196181

**Published:** 2024-09-24

**Authors:** Dan Tian, Xin Yan, Dong Zhou, Chen Wang, Wenshuai Zhang

**Affiliations:** Institute of Electronic Science and Technology, University of Electronic Science and Technology of China, Chengdu 611731, China; tiandan@uestc.edu.cn (D.T.); xinyan202101@163.com (X.Y.); wchen0227@163.com (C.W.); zhangwenshuaiii@163.com (W.Z.)

**Keywords:** dual-branch image object detection, IV-YOLO, bi-directional pyramid feature fusion, attention mechanism, small target detection

## Abstract

With the rapid growth in demand for security surveillance, assisted driving, and remote sensing, object detection networks with robust environmental perception and high detection accuracy have become a research focus. However, single-modality image detection technologies face limitations in environmental adaptability, often affected by factors such as lighting conditions, fog, rain, and obstacles like vegetation, leading to information loss and reduced detection accuracy. We propose an object detection network that integrates features from visible light and infrared images—IV-YOLO—to address these challenges. This network is based on YOLOv8 (You Only Look Once v8) and employs a dual-branch fusion structure that leverages the complementary features of infrared and visible light images for target detection. We designed a Bidirectional Pyramid Feature Fusion structure (Bi-Fusion) to effectively integrate multimodal features, reducing errors from feature redundancy and extracting fine-grained features for small object detection. Additionally, we developed a Shuffle-SPP structure that combines channel and spatial attention to enhance the focus on deep features and extract richer information through upsampling. Regarding model optimization, we designed a loss function tailored for multi-scale object detection, accelerating the convergence speed of the network during training. Compared with the current state-of-the-art Dual-YOLO model, IV-YOLO achieves mAP improvements of 2.8%, 1.1%, and 2.2% on the Drone Vehicle, FLIR, and KAIST datasets, respectively. On the Drone Vehicle and FLIR datasets, IV-YOLO has a parameter count of 4.31 M and achieves a frame rate of 203.2 fps, significantly outperforming YOLOv8n (5.92 M parameters, 188.6 fps on the Drone Vehicle dataset) and YOLO-FIR (7.1 M parameters, 83.3 fps on the FLIR dataset), which had previously achieved the best performance on these datasets. This demonstrates that IV-YOLO achieves higher real-time detection performance while maintaining lower parameter complexity, making it highly promising for applications in autonomous driving, public safety, and beyond.

## 1. Introduction

Dual-modality image detection is a key research area in computer vision that enhances the performance and robustness of object detection systems by integrating information from two perceptual modalities [1]. Traditional single-modality detection methods rely solely on information from one modality, making them susceptible to variations in lighting, occlusion, and environmental complexity, which can lead to reduced detection accuracy or increased false positive rates [2,3]. In contrast, dual-modality image detection overcomes these limitations, improving detection capabilities in complex scenarios [4].

Infrared images provide stable thermal signals in low-light, nighttime, or adverse weather conditions. Existing infrared detection frameworks, such as the slow-fast tubelet (SFT) [5] and the AIR-Net [6], have demonstrated significant advantages in low-light or dark environments by optimizing infrared data processing and feature fusion. In contrast, visible light images offer rich detail and color information under normal lighting conditions. For instance, the Depth Attention Enhancement Module (DAEM) and the RGB-Depth Fusion Module (RDFM) introduced in the MDFN model effectively extract fine texture details from visible light images [7], addressing issues like insufficient depth information and noise interference, which greatly improve detection performance. The fusion of infrared and visible light modalities significantly enhances the robustness of object detection. However, achieving an effective fusion of these two modalities presents a complex challenge, requiring the establishment of meaningful correlations and complementary relationships between them [8]. Due to the inherent differences between infrared and visible light modalities, the feature extraction and fusion process must overcome these disparities and fully leverage the unique strengths of each modality. Thus, ensuring feature complementarity between the two modalities is critical for successful fusion [9].

By thoroughly investigating the complementary nature of information between different sensing modalities, the model’s ability to capture richer and more diverse target features can be enhanced, leading to more accurate target recognition and classification. This approach improves detection accuracy and effectively reduces false positives and missed detections, equipping the model with greater adaptability to complex and dynamic real-world scenarios [10]. Furthermore, designing an appropriate network architecture and loss function is crucial to optimizing the model’s learning capacity and generalization performance, ensuring stable results across diverse environments [9].

Significant advancements have been made in dual-modal image object detection in recent years. For instance, FusionNet [11] utilizes Convolutional Neural Networks (CNNs) as a foundational feature extractor and inputs the fused feature maps into the object detection network to enhance detection performance. Dual-YOLO [12] introduces an information fusion module and a fusion shuffling module, enabling the network to complete infrared images using visible light features, thus improving detection accuracy and robustness. Guan et al. [13] extract features from infrared and visible light images separately using deep convolutional neural networks and integrate these features with a convolutional fusion module. Kim et al. [14] employed a joint training approach to enhance overall detection performance further. J. Zhu et al. [15] designed a Modal Interaction Module based on the Transformer architecture, which fuses features from RGB and thermal infrared images and incorporates a Query Location Module for precise object localization. Z Ye et al. [16] proposed the Cross-Modality Fusion Transformer, utilizing a Cross-Modality Fusion Transformer structure to effectively integrate information from both modalities through Transformer modules, thereby improving the performance of dual-modal drone object detection.

However, current dual-modal object detection methods still exhibit four main limitations. First, the precise control and interpretation of dual-modal feature fusion remain challenging, often resulting in either information redundancy or insufficiency. As noted by Gao et al. [17], although deep learning has advanced image fusion, the absence of well-designed loss functions can lead to suboptimal fusion performance. Ataman et al. [18] also point out that balancing preserving details with eliminating information redundancy across different spectral features remains problematic. This reflects the limitations of current methods in extracting and fusing both deep and shallow features, particularly in ensuring the sufficiency and effectiveness of the fused information. Second, the loss of deep information in complex scenes significantly affects detection accuracy, especially during the forward propagation of fusion networks. Zhao et al. [19] mitigate this issue by employing a dual-scale decomposition mechanism that processes low-frequency base information and high-frequency details separately, thereby reducing deep information loss.Similarly, Wang et al. [20] enhance the retention of background details and highlight infrared features by introducing multi-scale information through an improved generative adversarial network. Third, extracting fine-grained features and detecting multi-scale objects in complex backgrounds remain difficult. Liu et al. [21] emphasize the advantages of multi-scale fusion strategies in addressing occlusion issues in complex scenes. In contrast, Bao et al. [22] highlight the critical role of multi-scale processing in dealing with intricate backgrounds. Lastly, the high parameter count of fusion networks results in large model sizes and increased computational costs, negatively impacting real-time performance. Nousias et al. [23] explore model compression and acceleration techniques to reduce computational burdens and meet real-time inference requirements. Additionally, Poeppel et al. [24] stress that optimizing computational resource consumption and enhancing inference speed are key to achieving efficient real-time detection.

We propose a real-time object detection method based on the dual-branch fusion of visible and infrared images to address the aforementioned challenges. This approach effectively overcomes common issues in feature fusion, such as feature disparity, information redundancy, and model optimization difficulties, enabling efficient and accurate real-time object detection. Experimental results demonstrate that the proposed dual-branch detection method significantly improves the detection accuracy of multi-scale objects in complex environments while maintaining a low parameter count. The main contributions of this paper are summarized as follows:

(1) Building on the outstanding performance of YOLOv8 [25] in real-time object detection, we have developed a dual-branch network named IV-YOLO. This network consists of one branch dedicated to feature extraction from infrared images and another for feature extraction from visible light images. Additionally, a small object detection layer is incorporated into the neck network. This approach effectively addresses issues of background complexity and target feature occlusion inherent in single-modal image detection, enabling the extraction of fine-grained features of small objects. IV-YOLO significantly improves detection accuracy for multi-scale objects in complex backgrounds by maintaining a low parameter count and high frame rate.

(2) We propose a solution incorporating the Bi-Concat module, which employs a bidirectional pyramid structure for the weighted fusion of features extracted from infrared and visible light images at different levels. This approach effectively reduces redundant features and optimizes the fusion of features from the two modalities.

(3) We have designed the Shuffle-SPP structure, which uses Spatial Pyramid Pooling (SPP) to extract features at multiple scales, thereby enhancing the model’s ability to detect multi-scale objects. Subsequently, an efficient hierarchical aggregation mechanism is employed to merge features from different levels, further improving feature representation. This approach also emphasizes different parts of the features to enhance the Precision of object details and localization. The multi-level feature fusion, shuffling, and pooling operations effectively minimize information loss and retain more critical details, improving overall network performance. A loss function is also introduced to enhance the focus on small objects and accelerate the network’s convergence.

(4) Our method has achieved state-of-the-art results on the challenging KAIST [26] multispectral pedestrian dataset and the Drone Vehicle dataset [27]. Furthermore, experiments on the multispectral target detection dataset FLIR [28] further validate the effectiveness and generalizability of the algorithm.

The remainder of this paper is structured as follows: Section 2 describes related work pertinent to our network. In Section 3, we detail the network architecture and methodology. Section 4 presents our experimental details and results, comparing them with state-of-the-art networks to validate the effectiveness of our approach. In Section 5, we summarize the research content and experimental findings.

## 2. Related Work

This section briefly reviews classical deep learning methods for real-time object detection, summarizes the research progress in multi-modal image fusion techniques, and discusses the structures and applications of attention mechanisms.

### 2.1. Real-Time Object Detector

With the rapid advancement of deep learning, particularly convolutional neural networks (CNNs), real-time object detection technology has made significant strides in classifying and localizing objects under low-latency conditions. Modern real-time object detection methods primarily rely on deep learning models such as the YOLO (You Only Look Once) series, SSD [29] (Single Shot MultiBox Detector), and Fast R-CNN [30]. The YOLO series achieves object localization and classification through a single forward pass. At the same time, SSD directly predicts the classes and locations of multiple objects using a convolutional neural network, and Fast R-CNN detects objects by first generating candidate regions and then classifying and precisely localizing them.

Among these, the YOLO series has gradually become mainstream due to its efficient design. Versions like YOLOv1 [31], YOLOv2 [32], and YOLOv3 [33] have defined the classic detection architecture, which typically consists of three parts: the backbone, the neck, and the head. YOLOv4 [34] and YOLOv5 [35] introduced the CSPNet design to replace DarkNet, combined with data augmentation strategies, an enhanced Path Aggregation Network (PAN), and more diversified model scales. YOLOv6 [36] introduced BiC and SimCSPSPPF in the neck and backbone and adopted anchor-assisted training and self-distillation strategies. YOLOv7 [37] introduced the E-ELAN layer, while YOLOv8 proposed the C2f block for efficient feature extraction and fusion. YOLOv9 [38] further improved the architecture with the introduction of GELAN and incorporated PGI to enhance the training process. The latest YOLOv10 [39] introduced a continuous dual assignment method with NMS-free (Non-Maximum Suppression) training, offering competitive performance and the advantage of low inference latency.

### 2.2. Deep Learning-Based Multimodal Image Fusion

In deep learning-based multi-modal image fusion detection, substantial research has been conducted to explore this problem. Several studies have employed convolutional neural networks to extract features from different modalities and enhance detection accuracy and robustness through feature-level fusion. For instance, Chen et al. [40] investigated how multi-modal features can be effectively fused using CNNs to improve detection performance. Generative adversarial networks have also been widely used in multi-modal image fusion detection to boost model robustness and detection Precision further. Li et al. [41] proposed a multi-modal GAN architecture that effectively combines visible light and thermal imaging, leveraging GANs to fuse images from different sensors.

Researchers have also explored combining multi-task learning with weakly supervised and self-supervised learning to optimize multi-modal detection performance. For example, F. Pahde et al. [42] introduced an innovative fusion architecture for handling visible light and thermal images in few-shot learning scenarios. Meanwhile, FFD [43] proposed a self-supervised learning framework capable of performing fusion tasks without the need for paired images. Furthermore, various methods have been developed to improve multi-modal image fusion, such as TIRNet [44], which addresses redundancy in fused features; RISNet [45], which incorporates a mutual information minimization module to reduce redundant information; and PearlGAN [46], which introduces a top-down guided attention module and structured gradient alignment loss to refine the image encoding process and reduce local semantic ambiguity. These advancements highlight the diversity and cutting-edge developments in multi-modal image fusion detection, offering new directions for improving fusion effectiveness and detection performance.

### 2.3. Attention Mechanism

In deep learning, attention mechanisms have garnered increasing attention due to their ability to enable networks to focus on key features, prioritizing the most informative representations while suppressing irrelevant ones. Self-attention methods, for example, compute the weighted sum of all positions in an image to capture the context at a specific location. The SE [47] models the inter-channel dependencies through two fully connected layers, while NLNet [48] aggregates global context specific to each query position into its attention module. Building on NLNet and SENet, Gcnet [49] introduced the GC block, which maintains global context awareness while capturing inter-channel dependencies. CBAM [50] replaces global average pooling with global max pooling to enhance feature extraction, and GSoP Net [51] introduces global second-order pooling, improving performance at the cost of higher computational overhead. ECA-Net [52] significantly reduces the complexity of the SE model by generating channel weights through a one-dimensional convolution filter. The non-local (NL) module proposed by Wang et al. [53] generates an attention map by computing a correlation matrix between each spatial point in the feature map.

Shuffle Attention [54] and DANet [55] flexibly fuse local features and global dependencies by weighting and combining attention modules from different branches. FcaNet [56] interprets the relationship between GAP and the initial frequencies of the discrete cosine transform, using residual frequency selection to extract channel information, while WaveNet leverages discrete wavelet transform to capture channel features. OrthoNet [57] validates the effectiveness of orthogonal filters, attributing their performance largely to the orthogonality of DCT kernels. These studies offer diverse directions and theoretical support for applying attention mechanisms in deep learning.

## 3. Methods

### 3.1. Overall Network Architecture

**Figure 1:**
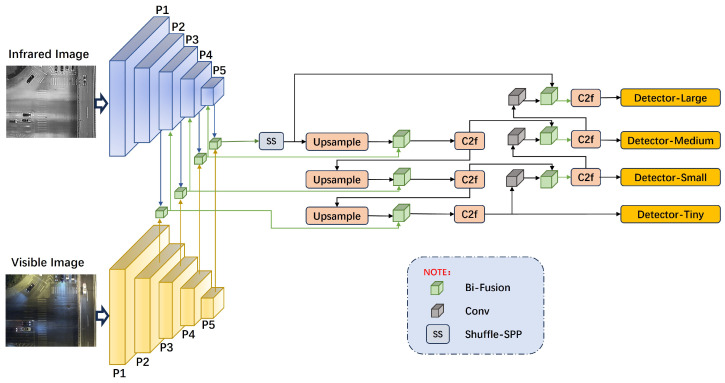
The overall architecture of the dual-branch IV-YOLO network. This network is specifically designed for object detection in complex environments. The backbone network extracts features from visible light and infrared images in parallel and fuses the multi-scale features obtained from the P2 to P5 layers. The fused features are fed into the infrared branch for deeper feature extraction. In the neck structure, we employ a Shuffle-SPP module, which integrates the extracted features with those from the backbone network through a three-layer upsampling process, enabling precise detection of objects at different scales.

**Figure 2:**
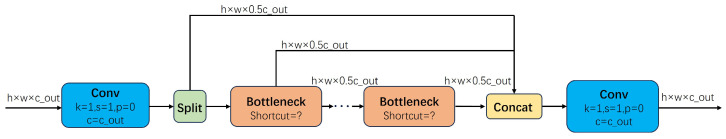
Diagram of the C2F Module.

We designed a dual-branch object detection network, IV-YOLO, based on the YOLOv8 framework, as illustrated in Figure 1. The network backbone consists of two branches that extract multi-scale object features from infrared and visible light images across five levels, from P1 to P5. At the P1 level, a single-layer convolutional (Conv) structure is employed, as defined by Equation (1). Here, $F_{C_{i}}\in R^{C_{in}\times H_{in}\times W_{in}}$, $F_{C_{i}}$ represents the input feature map, while conv denotes a convolution operation with a kernel size of 3 × 3 and a stride of 2. Batch normalization (BatchNorm2d) is applied to accelerate network convergence and enhance training stability, and the SiLU activation function ensures the non-linearity of the convolution operation. From P2 to P5, convolutional layers are integrated with the C2f structure proposed by YOLOv8, with the specific design of C2f shown in Figure 2.

(1) $$\mathrm{Conv}\left(F_{C_{i}}\right)=\mathrm{SiLU}\left(\mathrm{BatchNorm}2d\left({\mathrm{conv}}_{3\times 3,s=2}\right.\right.\left.\left.\left(F_{C_{i}}\right)\right)\right)$$

In the feature fusion phase, we have designed a novel Bidirectional Pyramid Fusion (Bi-Fusion) module detailed in Section 3.2.1. Under well-lit conditions, visible light images capture rich target details. In contrast, infrared images provide significant contrast in low-light environments and can penetrate certain materials for detection due to their thermal sensitivity. Thus, we employ the Bi-Fusion module to perform bidirectional pyramid fusion of features extracted from both branches at different scales (P2, P3, P4, P5). The P2 layer, with the smallest receptive field, is suited for fusing features of small targets; as the number of convolutional layers increases from P3 to P5, the receptive field expands, corresponding to the fusion of features for small, medium, and large targets. Experiments demonstrate that these fused features effectively complement the detailed information from the infrared branch. Consequently, we input the fused feature map vectors from layers P2 to P4 into layers P3 to P5 of the infrared branch for further feature extraction.

In the design of the neck structure, we introduced the Shuffled Spatial Pyramid Pooling (Shuffle-SPP) module. This module enhances feature utilization efficiency through multi-scale pooling, multi-layer convolution operations, and channel and spatial attention mechanisms. Its specific structure and characteristics are detailed in Section 3.2.2. Additionally, we incorporated elements from YOLOv8, adding an upsampling layer and a convolutional concatenation operation to retain multi-scale features and mitigate the gradual feature loss that can occur with convolutional operations, thereby enhancing object detection capability.

This architecture ensures comprehensive extraction of target texture details from visible light images and thermal radiation and material features from infrared images. Integrating multi-level feature fusion with shuffled attention mechanisms guarantees optimal utilization of dual-modal features, further improving the network’s robustness in complex environments.

### 3.2. Feature Fusion Structure

Our feature fusion structure comprises two key components: the Bi-Fusion structure and the Shuffle-SPP structure. The Bi-Fusion structure is primarily used to integrate multi-modal features from visible light and infrared images. In contrast, the Shuffle-SPP structure consolidates the extracted features within the deep network. By enhancing the relationships between channels, the Shuffle-SPP structure improves the network’s ability to focus on target features across different locations and channels. This design ensures effective feature fusion and enhances object detection performance in complex scenes.

#### 3.2.1. Bidirectional Pyramid Feature Fusion Structure

**Figure 3:**
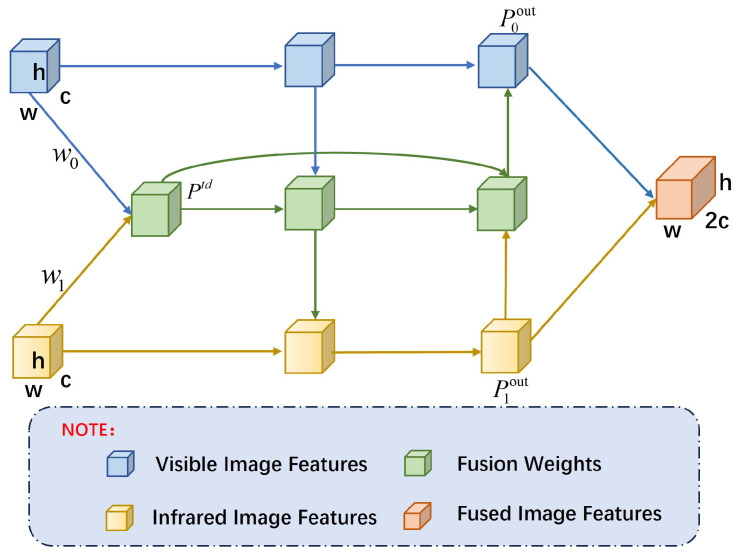
Bidirectional Pyramid Feature Fusion Structure, Bi-Fusion Structure.

We designed a Bi-Fusion structure in the feature fusion module to effectively merge features from infrared and visible light images. The Bi-Fusion structure enhances feature complementarity and information exchange between the infrared and visible light channels. Drawing on the design principles of EfficientDet, as illustrated in Figure 3, this structure integrates features extracted by both branches through bidirectional connections, which enhance the flow and fusion of features across different scales. Additionally, extra edges are introduced between input and output nodes at the same level, treating each bidirectional path as a feature network layer. This configuration optimizes cross-scale connections and improves the effectiveness of feature fusion.

To address the issue of uneven contributions from the infrared and visible light image branches to the output features under varying resolutions, lighting conditions, and backgrounds, we introduced learnable weights to optimize the feature fusion performance. This design enables the network to assess the importance of different input features effectively, thereby improving fusion efficacy. Figure 3 illustrates the weight allocation structure, where $w_{i}$ represents the weight assigned to input *i*. We employ the Fast Normalized Fusion method to obtain the fused features from the two modalities, as detailed in Equation (2).

(2) $$O=\sum_{i}\frac{w_{i}}{\epsilon +\sum_{j}w_{j}}\cdot I_{i}$$

The condition $w_{i}⩾0$ is ensured by applying a ReLU activation function after each $w_{i}$. The term ϵ=0.0001 is added to avoid division by zero and stabilize numerical computation. Equations (3)–(5) describe the weight parameter fusion of Bi-Fusion.

(3) $$P^{td}=\mathrm{Conv}\left(\frac{w_{0}\cdot P_{0}^{\mathrm{in}\;}+w_{1}\cdot P_{1}^{\mathrm{in}\;}}{w_{0}+w_{1}+\epsilon }\right)$$

(4) $$P_{0}^{\mathrm{out}\;}=\mathrm{Conv}\left(\frac{\left(w_{0}\cdot P_{0}^{\mathrm{in}\;}+w_{1}\cdot P^{td}\right)\cdot w_{0}}{\left(w_{0}+w_{1}\right)\cdot w_{0}+\epsilon }\right)$$

(5) $$P_{1}^{\mathrm{out}\;}=\mathrm{Conv}\left(\frac{\left(w_{1}\cdot P_{1}^{\mathrm{in}\;}+w_{0}\cdot P^{td}\right)\cdot w_{1}}{\left(w_{1}+w_{0}\right)\cdot w_{1}+\epsilon }\right)$$

In the above equation, $P_{0}^{\mathrm{in}\;}$ and $P_{1}^{\mathrm{in}\;}$ represent the features extracted from the infrared and visible light images, respectively, while $w_{0}$ and $w_{1}$ are the corresponding initial weight parameters. $P^{td}$ is the normalized intermediate feature generated by applying a weighted average to the two weighted inputs, followed by normalization and a convolution operation. $P_{0}^{\mathrm{out}\;}$ and $P_{1}^{\mathrm{out}\;}$ denote the fused output features for the infrared and visible light images, respectively. These are computed using the weight parameters, input features, and intermediate features described by Equations (4) and (5).

The Bi-Fusion structure achieves efficient feature flow and information exchange across different scales through bidirectional cross-scale connections and a repeated module design. This bidirectional flow promotes deep integration of features across scales, leveraging the complementarity of features from each scale while avoiding significant increases in computational cost. Compared with traditional unidirectional pyramid structures, Bi-Fusion maximizes feature utilization through bidirectional information flow, enhancing detection performance and reducing information loss between different feature levels. This approach helps the network capture more details, providing richer semantic and spatial information, particularly when dealing with targets of varying scales.

The synergistic effect of Bi-Fusion’s bidirectional connections and repeated module design enables feature complementarity between modalities, resulting in more diverse and expressive feature representations. Through multiple iterations of feature flow across different scales, the network progressively optimizes these features, better adapting to complex scenarios and accurately detecting targets of various sizes.

#### 3.2.2. Shuffle Attention Spatial Pyramid Pooling Structure

To enhance the capability of deep networks in learning mixed features from infrared and visible light images and to leverage the advantages of both modalities fully, we have introduced the Shuffle Attention mechanism [54]. This mechanism disrupts the constraints of local features through feature shuffling, promoting balanced information propagation across different spatial locations and channels, thus enhancing feature complementarity and flexibility in information flow. Compared with complex attention mechanisms such as self-attention in Transformers, Shuffle Attention offers lower computational complexity. Employing simple shuffling and local pooling operations significantly reduces computational costs while effectively facilitating information exchange, making it particularly suitable for large-scale data processing. Additionally, the integration of Shuffle Attention with Spatial Pyramid Pooling (SPP) enables multi-scale information fusion, assisting the network in capturing image features across various scales and enhancing global perception, which improves accuracy in detection and classification tasks.

**Figure 4:**
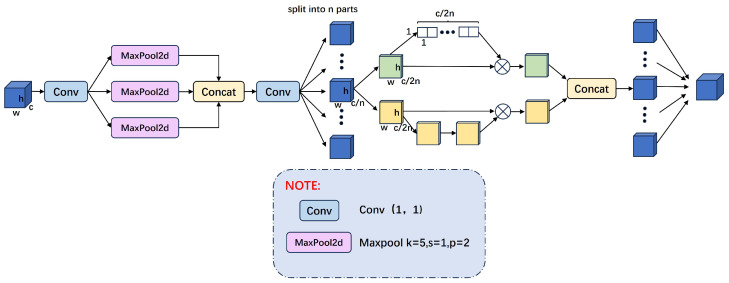
Shuffle Attention Spatial Pyramid Pooling Structure.

The overall structure of the designed Shuffle-SPP module is illustrated in the figure. After feature extraction and fusion through layers P1 to P5, the deepest fused features are input into the Shuffle-SPP module. This module employs multi-scale pooling, convolutional concatenation, and the Shuffle Attention mechanism to facilitate information exchange between the two modalities. Combined with efficient layer aggregation, it significantly enhances the model’s feature representation capability, allowing for the extraction of more comprehensive features. The overall structure of the Shuffle-SPP module is shown in Figure 4.

In Figure 4, we first adjust the number of channels using a Conv layer, followed by three MaxPool2d layers for multi-scale feature extraction. Next, we aggregate the extracted spatial information and restore the features through a Conv layer to maintain the original image dimensions. To better integrate the different characteristics of infrared and visible light modes, we divide the channels into n sub-features $X_{k}$ and iterate through each one. Each sub-feature $X_{k}$ is further divided into two parts: $X_{k1}$ and $X_{k2}$. The $X_{k1}$ part fully utilizes different channels. It applies the channel attention mechanism to focus on key features. In contrast, the $X_{k2}$ part leverages the spatial relationships of the features, using the spatial attention mechanism to highlight important features.

The design of the $X_{k1}$ part references the SE module and employs a channel attention mechanism. The module uses a single-layer transformation of GAP + Scale + Sigmoid to convert the feature $X_{k1}$ from dimensions $\frac{w\cdot h\cdot c}{2n}$ to 11c through global average pooling, as shown in Equations (6) and (7). Then, the obtained channel weights are normalized and multiplied with $X_{k1}$ to obtain the feature module $X_{k1}^{\prime }$ with channel attention. As training progresses, the module gradually captures specific semantic information.

(6) $$s=F_{gp}\left(X_{k1}\right)=\frac{1}{h\cdot w}\sum_{i=1}^{h}\sum_{j=1}^{w}X_{k1}\left(i,j\right)$$

(7) $$X_{k1}^{\prime }=\sigma \left(s\right)\cdot X_{k1}$$

The design of the $X_{k2}$ part employs a spatial attention mechanism, which, unlike channel attention, focuses on ‘where’ the information is, complementing channel attention. First, we use Group Norm (GN) [50] on $X_{k2}$ to obtain spatial statistics and apply a function to enhance the representation of $X_{k2}^{\prime }$. Subsequently, the obtained spatial weights are multiplied with $X_{k2}$ to produce the feature module $X_{k2}^{\prime }$ with spatial attention, as shown in Equation (8). As training progresses, the module gradually captures specific spatial information.

(8) $$X_{k2}^{\prime }=\sigma \left(W_{2}\cdot GN\left(X_{k2}\right)+b_{2}\right)\cdot X_{k2}$$

Then, all sub-features are aggregated to obtain a feature module with comprehensive information interaction.

The Shuffle-SPP module acquires global context information by rearranging and pooling features across the channel and spatial dimensions. It effectively combines relevant information from different channels while capturing a broader spatial context. This process allows the network to focus more precisely on the semantic and positional information of the target when processing deep features, thereby enhancing the model’s expressiveness and target detection accuracy. In this way, the Shuffle-SPP module enriches feature representation and strengthens the network’s ability to recognize targets in complex scenarios.

### 3.3. Loss Function

We categorize detection targets into four classes: micro, small, medium, and large. There is an imbalance in sample distribution among these categories, with a higher number of medium and large targets than micro and small targets. Medium and large targets are generally more adequately represented, allowing for better localization and detection. In contrast, precise localization of micro and small targets poses significant challenges. Consequently, we place particular emphasis on the performance of these hard-to-detect samples in bounding box regression and have enhanced the loss function based on PIoU v2.

Intersection over Union (*IoU*) is a metric used to assess the overlap between a predicted bounding box (*A*) and a ground truth bounding box (*B*). It calculates the ratio of their intersection to their union, as shown in Equation (9).

(9) $$IOU=\frac{\left(A\cap B\right)}{A\cup B}$$

To better handle the regression samples of different categories and focus on their respective detection tasks, we adopt a linear interval mapping method to reconstruct the IoU loss. This method enhances the regression accuracy for edge targets. The formula is as follows:

(10) $$IoU^{\mathrm{Focaler}\;}=\left\{\begin{array}{cc} 0, & IoU<d\\ \frac{IoU-d}{u-d}, & d⩽IoU⩽u\\ 1, & IoU>u \end{array}\right.$$

Here, $IoU^{\mathrm{Focaler}\;}$ represents the reconstructed Focaler-*IoU*, and *IoU* is the original value, [d,u]∈[0,1]. By adjusting the values of *d* and *u*, we can make $IoU^{\mathrm{Focaler}\;}$ focus on different regression samples. The loss is defined as follows:

(11) $$L_{\text{Focaler-}IoU\;}=1-IoU^{\mathrm{Focaler}\;}$$

By optimizing $L_{\text{Focaler-}IoU\;}$, the parameters of the feature extraction network can be improved, redundant image features can be eliminated, and the network’s generalization ability can be enhanced, thereby speeding up convergence.

To enhance the stability of bounding box regression, we used Complete *IoU* (CIOU) Loss as the loss function to address coordinate positioning errors. The calculation formula for CIOU is provided in Equation (12).

(12) $$CIOU=IOU-\frac{\rho^{2}\left(b,b^{g}\right)}{c^{2}}-\alpha \cdot v$$

where $\rho \left(b,b^{g}\right)$ denotes the distance between the center points of the predicted bounding box and the ground truth bounding box, *c* represents the diagonal length of the smallest enclosing box, α is the weight used to adjust the impact of the aspect ratio, and *v* measures the discrepancy in aspect ratio. We combined CIOU with $L_{\text{Focaler-}IoU}$ to create a loss function tailored for our network. The structure of this loss function is presented in Equation (13).

(13) $$L_{\text{Focaler-}CIoU\;}=L_{CIoU}+IoU-IoU^{\mathrm{Focaler}\;}$$

PIoU v2 simplifies the tuning process by requiring only one hyperparameter. It enhances focus on high-quality anchor boxes by combining a non-monotonic attention function with PIoU. Building on the PIoU loss, the PIoU v2 loss function introduces an attention layer that improves the model’s ability to focus on anchor boxes of moderate quality, thereby enhancing the performance of the object detector. By emphasizing the optimization of moderately high-quality anchor boxes during regression, PIoU v2 more effectively improves detection accuracy.

## 4. Results

In this chapter, we describe the implementation process for the dual-branch infrared and visible light image detection network, including hardware and software configuration specifics. To validate the effectiveness of the proposed method, we conducted extensive experiments across several publicly available datasets, including the Drone Vehicle dataset, the KAIST dataset, and the FLIR pedestrian dataset. The experimental results demonstrate that our method performs exceptionally well on these datasets.

### 4.1. Dataset Introduction

#### 4.1.1. Drone Vehicle Dataset

The Drone Vehicle dataset [27] is a publicly available resource specifically designed for drone-based object detection and classification tasks. It is extensively used in traffic monitoring and intelligent transportation system research. Drones capture the dataset and provide high-resolution aerial images covering various traffic scenarios, including urban roads, highways, and parking lots. The images are meticulously annotated with positional information, bounding boxes, and class labels for vehicles like cars, trucks, and buses. The dataset spans a range of environmental conditions from daytime to nighttime and includes infrared and visible light images, totallng 15,532 image pairs (31,064 images in total) and 441,642 annotated instances. Additionally, it accounts for real-world challenges such as occlusion and scale variation.

#### 4.1.2. FLIR Dataset

The FLIR dataset [28], released by FLIR Systems, is a publicly available resource widely used for infrared image object detection and pedestrian detection research. The dataset primarily consists of infrared images accompanied by corresponding visible light images, facilitating the exploration of multimodal fusion techniques. It covers a range of scenarios and environmental conditions, including daytime, nighttime, urban streets, and rural roads, to assess the robustness of detection algorithms under varying lighting conditions and complex backgrounds. The dataset includes over 10,000 pairs of 8-bit infrared images and 24-bit visible light images, encompassing targets such as people, vehicles, and bicycles. The resolution of the infrared images is 640 × 512 pixels, while the resolution of the visible light images ranges from 720 × 480 to 2048 × 1536 pixels.

#### 4.1.3. KAIST Dataset

The KAIST dataset [26], released by the Korea Advanced Institute of Science and Technology, is a publicly available resource extensively used for multimodal object detection and tracking research. This dataset includes visible light and infrared images, facilitating the study of information fusion across different modalities to enhance detection performance. It spans various scenarios and environmental conditions, including daytime, nighttime, sunny, and rainy conditions, which helps evaluate the robustness of algorithms under diverse lighting and weather conditions. The dataset focuses on pedestrian detection and provides detailed annotation information, including pedestrians’ location, size, and category labels. The dataset is divided into 12 subsets: sets 00 to 05 are used for training (sets 00 to 02 for daytime scenes and sets 03 to 05 for nighttime scenes), and sets 06 to 11 are used for testing (sets 06 to 08 for daytime scenes and sets 09 to 11 for nighttime scenes). The images have a 640 × 512 pixels resolution and include 95,328 images, each containing visible light and infrared. The KAIST dataset encompasses a range of typical traffic scenarios, including campus, street, and rural environments, with 103,108 densely annotated objects.

In our experiments, we resized each visible image to 640 × 640. Table 1 summarizes the dataset information used for training and testing.

### 4.2. Implementation Details

We implemented the code using the PyTorch (version 1.12.1) framework and conducted experiments on a workstation with an NVIDIA RTX 4090 GPU. Details of the experimental environment and parameter settings are provided in Table 2, while the hyperparameters for the datasets are listed in Table 3. To ensure training and testing accuracy, we maintained a consistent number of infrared and visible light images and performed data cleaning during the network training and testing phases.

During data augmentation, each image had a 50% chance of undergoing random horizontal flipping. Additionally, we employed mosaic operations to stitch multiple images into a single composite, enhancing the complexity and diversity of the training samples to improve the model’s adaptability to different scenes and viewpoints. The entire network was optimized using the AdamW optimizer, combined with weight decay, and trained for 300 epochs. The learning rate was set to 0.001, with a batch size of 32, weight decay of 0.0005, and momentum warm-up set to 0.8. These hyperparameters were chosen based on the specific challenges and characteristics of the datasets: a lower learning rate facilitates more precise model parameter adjustments for high-resolution images, weight decay reduces the risk of overfitting, a batch size of 32 balances memory usage and training stability, and momentum warm-up accelerates convergence in the early stages of training. The AdamW optimizer, which integrates the advantages of the Adam optimizer while effectively mitigating overfitting through weight decay, is particularly well-suited for handling complex multimodal datasets such as Drone Vehicle, FLIR, and KAIST, thereby providing more stable and efficient training and enhancing detection performance and accuracy.

### 4.3. Evaluation Metrics

In this study, we evaluate the detection performance of the network using Precision, Recall, and mean Average Precision (mAP). Additionally, we assess the network’s efficiency by considering the number of parameters and Frames Per Second (FPS).

Precision and Recall are primarily used during the experiments to measure the network’s performance, with the calculations shown in Equations (14) and (15).

(14) $$precision=\frac{\mathrm{TP}}{\mathrm{TP}+\mathrm{FP}}$$

(15) $$recall=\frac{\mathrm{TP}}{\mathrm{TP}+\mathrm{FN}}$$

Specifically, TP (True Positive) represents the number of correctly predicted positive samples, FP (False Positive) denotes the number of incorrectly predicted positive samples, and FN (False Negative) refers to the number of positive samples that were incorrectly predicted as negative. Average Precision (AP) is the area under the Precision–Recall curve, and the closer the AP value is to 1, the better the detection performance of the algorithm.

(16) $$\mathrm{AP}=\int_{0}^{1}p\left(r\right)dr$$

Mean Average Precision (mAP) is the average of the AP values across all classes, offering a balanced evaluation by combining Precision and Recall. In multi-class detection tasks, mAP is particularly important because it ensures good performance across all classes. Moreover, mAP is robust to class imbalance, making it widely used in evaluating multi-class object detection tasks. It is also a key metric in our experiments to assess detection accuracy. The formula for calculating mAP is as follows:

(17) $$\mathrm{mAP}=\frac{1}{C}\sum_{c=1}^{C}\mathrm{AP}$$

The number of parameters and FPS evaluate the efficiency of the model. The number of parameters refers to the total count of all learnable parameters, including weights and biases. FPS measures how many image frames the network can process per second, an essential real-time performance indicator.

### 4.4. Analysis of Results

This section tests the IV-YOLO network on three test datasets and compares the detection results with advanced methods.

#### 4.4.1. Experiment on the DroneVehicle Dataset

We conducted a series of experiments on the Drone Vehicle dataset to verify the proposed dual-branch object detection method’s capability in detecting small objects in complex environments. During the experiments, the dataset was preprocessed by cropping 100 pixels from each edge of the images to remove the white borders, resulting in images of size 640 × 512. The detection head was also replaced with an oriented bounding box (OBB) detection head.

In the Drone Vehicle dataset, the shapes of freight cars and vans are quite similar, and many existing detection methods often omit these two categories to avoid fine-grained classification errors. However, our experiments used the complete Drone Vehicle dataset to assess the network’s ability to extract and fuse fine-grained dual-modal features. Table 4 presents a comparative analysis of our method’s performance against other networks.

Since many network models are based on single-modal images for detection, we evaluated these networks using visible and infrared images. As shown in Table 4, YOLOv8 demonstrates outstanding performance in detection accuracy and speed in single-modal scenarios, so we chose YOLOv8 as the foundational framework for the IV-YOLO algorithm. Through our innovative improvements, the IV-YOLO algorithm achieved an accuracy of 74.6% on the Drone Vehicle dataset, outperforming all other networks. Notably, in fine-grained feature extraction and fusion, IV-YOLO achieved detection accuracies of 63.1% for freight cars and 53% for vans, significantly surpassing other networks.

The results in Table 4 indicate that IV-YOLO effectively integrates dual-modal features to enable robust detection in complex environments and improves the accuracy of detecting visually similar objects through dual-branch feature fusion. However, the emphasis on fine-grained features led to a decrease in detection performance for visually similar categories. Additionally, in small object detection, the network focuses more on extracting low-level features to capture fine details, as small objects require higher-resolution feature maps and precise local details. This focus may weaken the network’s performance on larger objects, as detecting larger targets requires a broader receptive field to understand the global context fully. An overemphasis on details can reduce the utilization of high-level semantic information, affecting larger objects’ detection performance.

**Figure 5:**
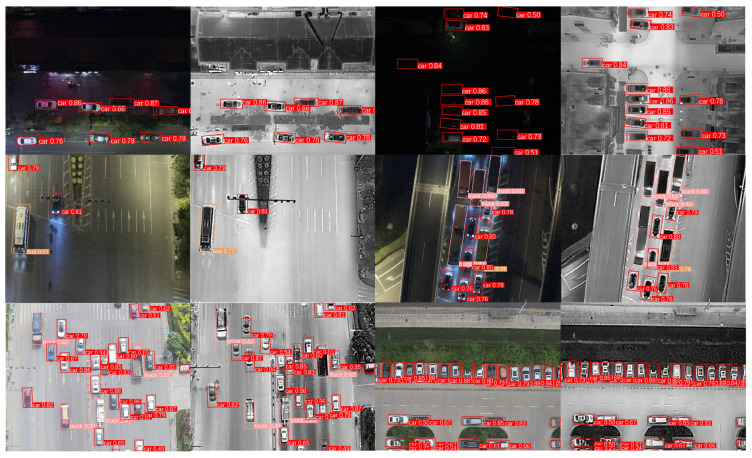
Visualization of IV-YOLO Detection Results Based on the Drone Vehicle Dataset.

Figure 5 illustrates the visualized detection results on the Drone Vehicle dataset. The images are divided into six groups, each containing three rows of images. The visible light detection results are on the left side of each group, while the right side shows the infrared image detection results. The first row demonstrates that the IV-YOLO network is capable of robust target detection even in low-light conditions or when soft occlusion is present. The second row highlights the network’s ability to effectively extract fine-grained features, successfully distinguishing between visually similar objects. The third row shows the network’s robustness when processing scenes with dense targets.

#### 4.4.2. Experiments on the FLIR Dataset

We conducted a series of experiments on the FLIR dataset to validate the effectiveness of the proposed method. We compared the performance of several detection algorithms, including SSD, YOLOv9, YOLOv10, and YOLOF, and evaluated them against the Dual-YOLO network, which has demonstrated outstanding performance on the FLIR dataset. The detailed results of these experiments are presented in Table 5.

The experimental results on the FLIR dataset are summarized in Table 6. The table shows that our network achieves the highest mAP value on this dataset, outperforming other methods. Integrating multi-scale feature fusion and the triple upsampling operations in the neck significantly enhances our network’s ability to extract features from small objects. The results indicate a noticeable improvement in Precision for detecting small objects, such as bicycles, which are challenging to extract. However, due to a slight reduction in global feature capture, the detection performance for larger objects, such as cars and pedestrians, is marginally lower than the Dual-YOLO network. Overall, the mAP results demonstrate that our network effectively extracts multi-scale features, particularly capturing fine details at smaller receptive field levels, thus improving the detection of small targets. Additionally, through the fusion module, our network effectively extracts and integrates shared features from both modalities and their unique characteristics. Through weighted parameters, the fusion mechanism enables mutual enhancement and compensation between the two modalities, leading to superior detection performance.

**Figure 6:**
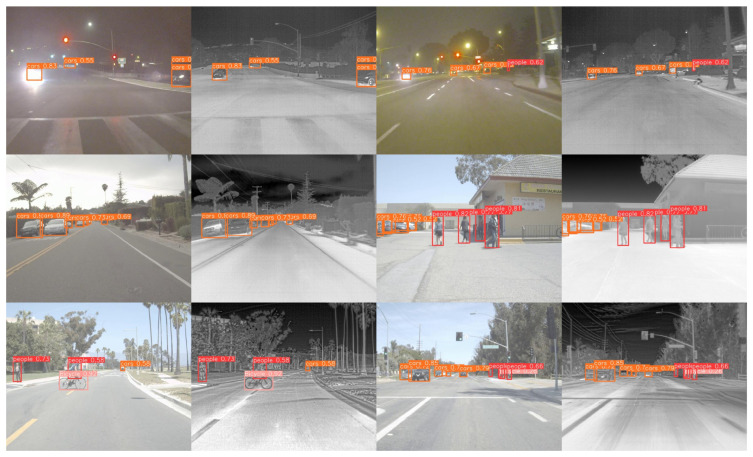
Visualization of IV-YOLO Detection Results on the FLIR Dataset.

Figure 6 illustrates the visualization of object detection results on the FLIR dataset. The first row of images demonstrates that, regardless of changes in background lighting, our network accurately locates and detects objects by integrating features from both modalities. The second row shows that the network effectively uses the dual-branch structure to extract fine-grained features of objects at different scales. Combined with a specially designed loss function, it successfully detects each object, even in cases of occlusion and high target density. The third row highlights the network’s advantage in multi-scale feature extraction, particularly at smaller receptive field levels, where it captures more subtle features, thus significantly enhancing the detection of small targets.

#### 4.4.3. Experiments Based on the KAIST Dataset

To further validate the effectiveness and robustness of our proposed IV-YOLO algorithm, we conducted experiments on the challenging KAIST dataset. Given numerous blank and misaligned images in the KAIST dataset, we first performed data cleaning and then performed the object detection task on the processed dataset. After comparing our results with several popular methods, our experimental outcomes are presented in Table 6.

As shown in Table 6, the performance of the network we proposed on the KAIST dataset significantly outperforms that of the first three methods, which exhibit notably lower accuracy. This discrepancy primarily arises because the training process for the KAIST dataset did not include data preprocessing, resulting in blank images and misaligned labels that impacted the accuracy of the results. After preprocessing the dataset, we compared our network with the state-of-the-art Dual-YOLO and PearlGAN methods. The results, detailed in the last three rows of Table 6, demonstrate that our method excels in Precision, Recall, and mAP.

**Figure 7:**
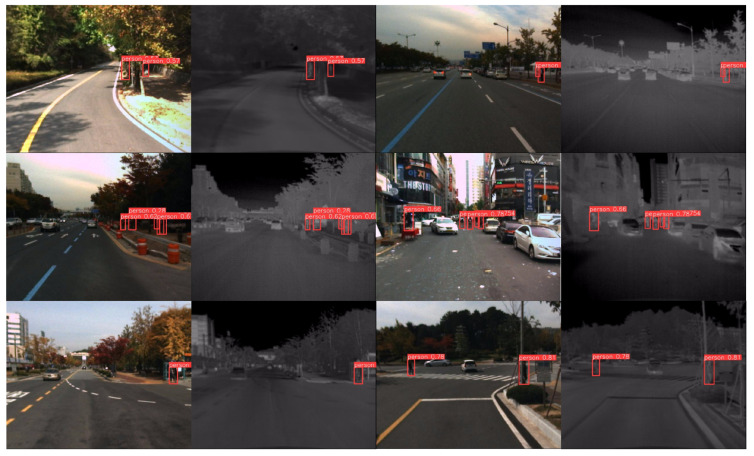
Visualization of IV-YOLO Detection Results on the KAIST Pedestrian Dataset.

Figure 7 presents the visualized test results of our network on the KAIST dataset. The figure includes six groups, with a total of twelve images. The first row illustrates the strong robustness of our network under varying scales and lighting conditions. The second row highlights our IV-YOLO network’s capability to detect pedestrians on the street despite overlapping and occlusions accurately. The third row demonstrates that, through feature fusion, our network effectively identifies targets even in complex backgrounds.

### 4.5. Parameter Analysis

In this study, we evaluated the proposed model’s parameter efficiency and inference speed on the Drone Vehicle and FLIR datasets. All tests were conducted on an NVIDIA RTX 3090 GPU (NVIDIA Corporation, Santa Clara, CA, USA), with the model parameters measured in megabytes (MB) using 16-bit Precision to ensure the accuracy of the experimental results. As shown in Table 7, the IV-YOLO network maintains high detection accuracy while keeping the parameter count low. Consequently, the model can be flexibly deployed on resource-constrained devices without compromising performance. When running on the NVIDIA RTX 3090, IV-YOLO achieved a real-time processing speed of up to 203 frames per second (FPS). This high FPS value highlights the model’s ability to perform fast and accurate object detection with high-resolution inputs. It is particularly suitable for applications where both speed and quality are critical. Green indicates the optimal results on the corresponding dataset.

### 4.6. Ablation Study

We designed and conducted a series of ablation experiments to thoroughly analyze the individual contributions of each module and component in the proposed model. By systematically removing key components from the network, we evaluated their impact on overall performance, thus clarifying the role of each module within the model. The experiments employed mAP@0.5 to assess model accuracy and mAP@0.5:0.95 as a comprehensive evaluation metric to eliminate the influence of varying *IoU* threshold settings on the results. The consequences ensured the completeness and fairness of the performance assessment.

First, we conducted ablation experiments on the feature fusion module. The Shuffle-SPP component, designed to enhance the internal correlation between deep features and improve the model’s ability to recognize and localize objects, was initially removed. After re-running the experiments on the standard dataset without this component, the model’s mAP dropped by 1.5%. This result demonstrates that including Shuffle-SPP effectively improves the extraction of deep features.

Next, a similar ablation experiment was performed on the Bi-Fusion module. Compared with removing Shuffle-SPP, eliminating Bi-Fusion had a more significant impact on overall model performance, with the mAP decreasing to 82.9%. This marked decline indicates that the Bi-Fusion structure is critical in effectively merging features from visible and infrared images and is essential for improving the model’s performance. The results of the ablation experiments for the feature fusion module are presented in Table 8. In this table, “
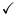
” indicates that the network includes the module, while “×” denotes that the module is not utilized.

**Figure 8:**
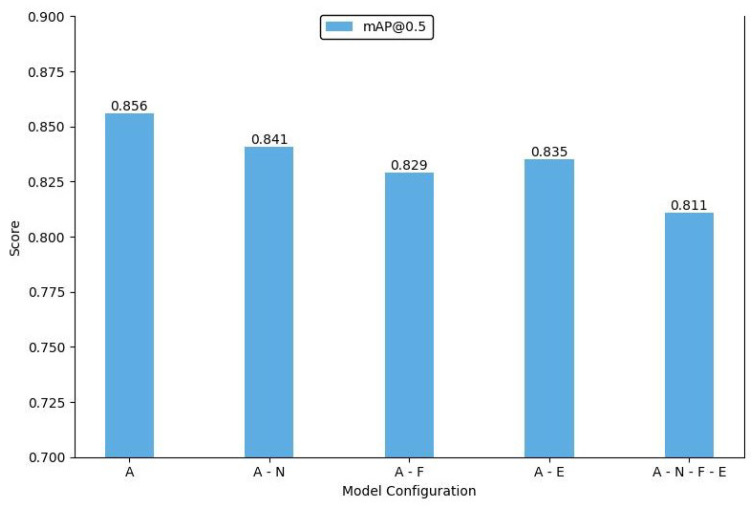
Bar chart of mAP@0.5 in the ablation experiments.

**Figure 9:**
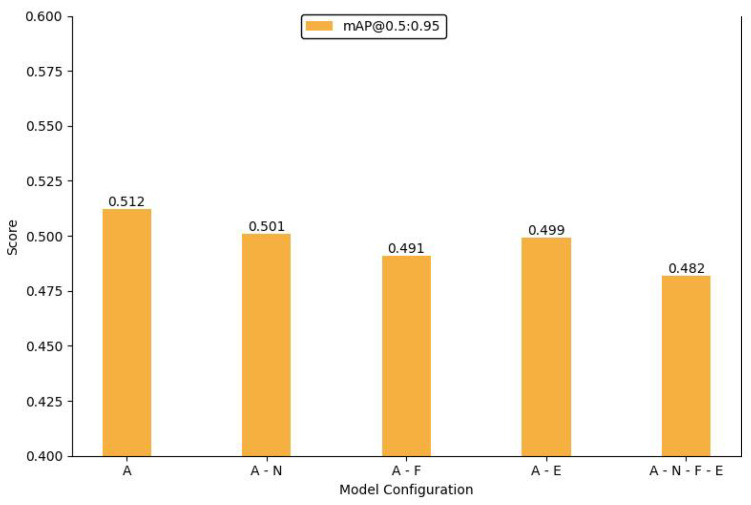
Bar chart of mAP@0.5:0.95 in the ablation experiments.

We also conducted experiments by removing an upsampling layer and a concatenation structure from the network. These components were specifically optimized for small object detection. Removing these elements resulted in significant performance fluctuations in detecting small objects, and there was a noticeable decline in the model’s ability to detect high-similarity objects. To fully assess these additional structures’ contribution, we removed all three components and analyzed their combined effect on model performance. The final ablation results are illustrated in Figure 8 and Figure 9. In these figures, ‘A’ represents our IV-YOLO network, ‘N’ denotes the Shuffle-SPP structure, ‘F’ indicates the Bi-Fusion structure, and ‘E’ refers to the network optimized for small object detection.

These ablation experiments reveal the crucial roles of each module and component within the model. By examining the impact of each component individually, we validated their importance for specific tasks, further demonstrating the rationality and effectiveness of the model’s design. The experimental results indicate that these modules exhibit significant complementarity across different tasks and scenarios. The combined use of these modules maximizes the model’s performance, while the absence of any single module markedly undermines the overall effectiveness of the model.

## 5. Conclusions

This paper presents a dual-branch object detection network based on YOLOv8, named IV-YOLO, which integrates infrared and visible light images. The network is meticulously designed to effectively extract features from both modalities during the dual-branch feature extraction process and perform object detection at multiple scales. Additionally, we developed a Bidirectional Pyramid Feature Fusion structure (Bi-Fusion) that integrates features from different modalities across multiple scales using weighted parameters. This approach effectively reduces the number of parameters and avoids feature redundancy, enhancing the network’s fusion performance. To further bolster the network’s expressive capability, we introduced the Shuffle Attention Spatial Pyramid Pooling structure (Shuffle-SPP), which captures global contextual information across channels and spatial dimensions. This structure enables the network to focus more accurately on the semantic and positional information of objects within deep features, significantly improving the model’s representational power. Finally, by incorporating the PIoU v2 loss function, we accelerated the convergence of object detection boxes for targets of varying sizes, enhanced the fitting Precision for small targets, and sped up the overall convergence of the network. Experimental results demonstrate that the IV-YOLO network achieves a mean Average Precision (mAP) of 74.6% on the Drone Vehicle dataset, 75.4% on the KAIST dataset, and 85.6% on the FLIR dataset. These results indicate that IV-YOLO excels in feature extraction, fusion, and object detection for infrared and visible light images. Furthermore, the network’s parameter count is only 4.31 M, significantly lower than other networks, showcasing its potential for deployment on mobile devices.

Despite its excellent performance in dual-modal object detection tasks, IV-YOLO has some limitations: (1) it requires further optimization of speed and memory usage before hardware deployment; (2) there is limited availability of dual-modal datasets with diverse scenes and targets, necessitating further research and validation of the method’s effectiveness; and (3) while the network enhances detection of small targets, future improvements are needed to strengthen its global perception capability. Although single-modal detection performs well for certain specific tasks, its limitations become evident in complex real-world applications. In contrast, dual-modal detection leverages the advantages of multiple data sources to provide richer feature information, significantly improving object detection accuracy and robustness. Therefore, dual-modal detection technology is undoubtedly a key development direction for future object detection research, offering strong support for addressing more complex detection tasks.

## Figures and Tables

**Table 1:** Dataset information we used in this paper.

Hyper Parameter	Drone Vehicle Dataset	FLIR Dataset	KAIST Dataset
Scenario	drone	adas	pedestrian
Modality	Infrared + Visible	Infrared + Visible	Infrared + Visible
Images	56,878	14,000	95,328
Categories	5	4	3
Labels	190.6 K	14.5 K	103.1 K
Resolution	840 × 712	640 × 512	640 × 512

**Table 2:** Experimental Setup and Parameter Settings.

Category	Parameter
CPU Intel	i7-12700H (Intel Corporation, Santa Clara, CA, USA)
GPU	NVIDIA RTX 4090 GPU (NVIDIA Corporation, Santa Clara, CA, USA)
System	Windows11
Python	3.8.19
PyTorch	1.12.1
Training Epochs	300
Learning Rate	0.01
Weight Decay	0.0005
Momentum	0.937

**Table 3:** The hyperparameters of the datasets used in this paper. ‘test-val’ indicates that the same dataset is used for both testing and validation in this study.

Hyper Parameter	DroneVehicle Dataset	FLIR Dataset	KAIST Dataset
Visible Image Size	640 × 640	640 × 640	640 × 640
Infrared Image Size	640 × 640	640 × 640	640 × 640
Visible Image	28,439	8437	8995
Infrared Image	28,439	8437	8995
Training set	17,990	7381	7595
Validation set	1469	1056	1400
Testing set	8980	1056 (test-val)	1400 (test-val)

**Table 4:** Evaluation results based on the Drone Vehicle dataset. All values are expressed as percentages. The top-ranked results are highlighted in green.

Method	Modality	Car	Freight Car	Truck	Bus	Van	mAP
RetinaNet (OBB) [58]	Visible	67.5	13.7	28.2	62.1	19.3	38.2
Faster R-CNN (OBB) [30]	Visible	67.9	26.3	38.6	67.0	23.2	44.6
Faster R-CNN (Dpool) [59]	Visible	68.2	26.4	38.7	69.1	26.4	45.8
Mask R-CNN [60]	Visible	68.5	26.8	39.8	66.8	25.4	45.5
Cascade Mask R-CNN [61]	Visible	68.0	27.3	44.7	69.3	29.8	47.8
RoITransformer [10]	Visible	68.1	29.1	44.2	70.6	27.6	47.9
YOLOv8n (OBB) [25]	Visible	97.5	42.8	62.0	94.3	46.6	68.6
Oriented RepPoints [62]	Visible	73.7	30.3	45.4	73.9	36.1	51.9
RetinaNet (OBB) [58]	Infrared	79.9	28.1	32.8	67.3	16.4	44.9
Faster R-CNN (OBB) [30]	Infrared	88.6	35.2	42.5	77.9	28.5	54.6
Faster R-CNN (Dpool) [59]	Infrared	88.9	36.8	47.9	78.3	32.8	56.9
Mask R-CNN [60]	Infrared	88.8	36.6	48.9	78.4	32.2	57.0
Cascade Mask R-CNN [61]	Infrared	81.0	39.0	47.2	79.3	33.0	55.9
RoITransformer [10]	Infrared	88.9	41.5	51.5	79.5	34.4	59.2
YOLOv8n (OBB) [25]	Infrared	97.1	38.5	65.2	94.5	45.2	68.1
Oriented RepPoints [62]	Infrared	87.1	39.7	50.1	77.6	36.9	58.3
UA-CMDet [27]	Visible + Infrared	87.5	46.8	60.7	87.1	38.0	64.0
Dual-YOLO [12]	Visible + Infrared	98.1	52.9	65.7	95.8	46.6	71.8
YOLOFIV [63]	Visible + Infrared	95.9	34.6	64.2	91.6	37.2	64.7
IR-YOLO(Ours)	Visible + Infrared	97.2	63.1	65.4	94.3	53.0	74.6

**Table 5:** The object detection results on the FLIR dataset, calculated at a single *IoU* threshold of 0.5. All values are expressed as percentages, with the top-ranked results highlighted in green.

Method	Person	Bicycle	Car	mAP
Faster R-CNN [30]	39.6	54.7	67.6	53.9
SSD [29]	40.9	43.6	61.6	48.7
RetinaNet [58]	52.3	61.3	71.5	61.7
FCOS [64]	69.7	67.4	79.7	72.3
MMTOD-UNIT [27]	49.4	64.4	70.7	61.5
MMTOD-CG [27]	50.3	63.3	70.6	61.4
RefineDet [65]	77.2	57.2	84.5	72.9
TermalDet [27]	78.2	60.0	85.5	74.6
YOLO-FIR [66]	85.2	70.7	84.3	80.1
YOLOv3-tiny [33]	67.1	50.3	81.2	66.2
IARet [67]	77.2	48.7	85.8	70.7
CMPD [68]	69.6	59.8	78.1	69.3
PearlGAN [46]	54.0	23.0	75.5	50.8
Cascade R-CNN [61]	77.3	84.3	79.8	80.5
YOLOv5s [35]	68.3	67.1	80.0	71.8
YOLOF [69]	67.8	68.1	79.4	71.8
YOLOv10n [39]	62.9	69.9	86.2	73.0
Dual-YOLO [12]	88.6	66.7	93	84.5
IV-YOLO (Ours)	86.6	77.8	92.4	85.6

**Table 6:** The object detection results on the KAIST dataset, calculated at a single *IoU* threshold of 0.5. All values are expressed as percentages, with the top-ranked results highlighted in green.

Method	Precision	Recall	mAP
ForkGAN [70]	33.9	4.6	4.9
ToDayGAN [71]	11.4	14.9	5.0
UNIT [72]	40.9	43.6	11.0
PearlGAN [46]	21.0	39.8	25.8
YOLOv9m [38]	76.5	40.5	60.8
YOLOv10n [39]	71.5	43.2	52.5
Dual-YOLO [12]	75.1	66.7	73.2
IV-YOLO(OURS)	77.2	84.5	75.4

**Table 7:** Model complexity and runtime comparison of IV-YOLO and the plain counterparts.

Method	Dataset	Params	Runtime (fps)
Faster R-CNN (OBB) [30]	Drone Vehicle	58.3 M	5.3
Faster R-CNN (Dpool) [59]	Drone Vehicle	59.9 M	4.3
Mask R-CNN [60]	Drone Vehicle	242.0 M	13.5
RetinaNet [59]	Drone Vehicle	145.0 M	15.0
Cascade Mask R-CNN [61]	Drone Vehicle	368.0 M	9.8
RolTransformer [10]	Drone Vehicle	273.0 M	7.1
YOLOv7 [37]	Drone Vehicle	72.1 M	161.0
YOLOv8n [25]	Drone Vehicle	5.92 M	188.6
IV-YOLO (Ours)	Drone Vehicle	4.31 M	203.2
SSD [29]	FLIR	131.0 M	43.7
FCOS [64]	FLIR	123.0 M	22.9
RefineDet [65]	FLIR	128.0 M	24.1
YOLO-FIR [66]	FLIR	7.1 M	83.3
YOLOv3-tiny [33]	FLIR	17.0 M	66.2
Cascade R-CNN [61]	FLIR	165.0 M	16.1
YOLOv5s [35]	FLIR	14.0 M	41.0
YOLOF [69]	FLIR	44.0 M	32.0
Dual-YOLO [12]	FLIR	175.1 M	62.0
IV-YOLO (Ours)	FLIR	4.91 M	194.6

**Table 8:** Ablation experiments of the feature fusion structure. All values are expressed as a percentage.

Shuffle-SPP	Bi-Concat	Person	Bicycle	Car	mAP@0.5	mAP@0.5:0.95
×	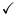	83.7	76.4	92.1	84.1	50.1
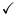	×	83.2	73.8	91.6	82.9	49.1
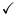	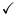	86.6	77.8	92.4	85.6	51.2

## Data Availability

The DroneVehicle remote sensing dataset is obtained from https://github.com/VisDrone/DroneVehicle, accessed on 29 December 2021. The KAIST pedestrian dataset is obtained from https://github.com/SoonminHwang/rgbt-ped-detection/tree/master/data, accessed on 12 November 2021. The FLIR dataset is obtained from https://www.flir.com/oem/adas/adas-dataset-form/, accessed on 19 January 2022.
